# Sleep disturbances in the context of neurohormonal dysregulation in patients with bipolar disorder

**DOI:** 10.1186/s40345-022-00254-8

**Published:** 2022-03-01

**Authors:** Tom Roloff, Ida Haussleiter, Klara Meister, Georg Juckel

**Affiliations:** Department of Psychiatry, Psychotherapy and Preventive Medicine, LWL University Hospital, Ruhr University Bochum, Alexandrinenstr. 1, 44791 Bochum, Germany

**Keywords:** Bipolar disorder, Sleep, Actigraphy, Circadian rhythm, Melatonin, DLMO, PAD, Cortisol

## Abstract

**Background:**

Sleep dysfunction is a core symptom in bipolar disorder (BD), especially during major mood episodes. This study investigated the possible link between subjective and objective sleep disturbances in inter-episode BD, changes in melatonin and cortisol levels, and circadian melatonin alignment. The study included 21 euthymic BD patients and 24 healthy controls. Participants had to wear an actigraphy device, keep a weekly sleep diary and take salivary samples: five samples on the last evening to determine the dim light melatonin onset (DLMO) and one the following morning to measure rising cortisol. Sleep quality was assessed by the Pittsburgh Sleep Quality Index (PSQI) and Regensburg Insomnia Scale (RIS), and circadian alignment by the phase angle difference (PAD).

**Results:**

In comparison to healthy controls, BD patients had: (1) higher PSQI (5.52 ± 3.14 vs. 3.63 ± 2.18; *p* = 0.022) (significant after controlling for age and gender), and higher RIS scores (8.91 ± 5.43 vs. 5.83 ± 3.76; *p* = 0.031); (2) subjective a longer mean TST (*p* = 0.024) and TIB (*p* = 0.002) (both significant after controlling for age and gender), longer WASO (*p* = 0.019), and worse SE (*p* = 0.036) (significant after controlling for gender); (3) actigraphically validated earlier sleep onset (*p* = 0.002), less variation in sleep onset time (*p* = 0.005) and no longer TST (*p* = 0.176); (4) no differing melatonin levels (4.06 ± 2.77 vs. 3.35 ± 2.23 *p* = 0.352), an 1.65 h earlier DLMO (20.17 ± 1.63 vs. 21.82 ± 1.50; *p* = 0. 001) (significant after controlling for gender), and a phase advance of melatonin (6.35 ± 1.40 vs. 7.48 ± 1.53; *p* = 0.017) (significant after controlling for gender); and (5) no differing cortisol awakening response (16.97 ± 10.22 vs 17.06 ± 5.37 *p* = 0.969).

**Conclusions:**

Patients with BD, even in euthymic phase, have a significantly worse perception of their sleep. Advanced sleep phases in BD might be worth further investigation and could help to explain the therapeutic effects of mood stabilizers such as lithium and valproate.

## Background

Bipolar disorder (BD) is a chronic, severe, and recurring mood disorder that may also affect cognitive performance, and circadian rhythm (American Psychiatric Association 2013). BD affects an estimated 1–2% of the population and its disability burden has been ranked one of the leading neuropsychiatric conditions in the WHO’s Global Burden of Disease Study (2022). Sleep disturbances (SD) are frequently associated with BD and bipolar episodes comprise mania with decreased need for sleep, depression with hyper- or insomnia, and mixed episodes displaying both traits (American Psychiatric Association 2013). SD often precede BD onset and have been regarded as prodromal symptoms in patients at risk (Pancheri et al. 2019; Ritter et al. 2011; Zeschel et al. 2013), which can affect the course of illness, quality of life, functioning, symptom burden, and overall treatment outcomes (Bradley et al. 2017; Harvey et al. 2015; Jermann et al. 2021). SD are also likely precursors of new affective episodes (Sylvia et al. 2012). Sleep of BD patients is not only altered during major mood episodes, but also in euthymic (or remitted) phases (Sylvia et al. 2012; Steinan et al. 2016) and substantially differing compared to healthy controls (Harvey et al. 2015; Kanady et al. 2015; Tazawa et al. 2019). Meta-analyses have proven BD—even in euthymic stages—to be associated with subjectively worse sleep quality (Ng et al. 2015), longer total sleep time (TST), longer sleep onset latency (SOL), and longer waking time after sleep onset (WASO); furthermore, SOL and WASO were followed by worse sleep efficiency (SE) (Geoffroy et al. 2015; Meyer et al. 2020). The mechanisms of the circadian rhythm are of great interest in BD, since this rhythm often seems unstable or disturbed (delayed or fragmented) (Bradley et al. 2017; Jones et al. 2005) and circadian rhythm dysfunction has been considered a trait marker of BD (Takaesu 2018). Melatonin as a neuroendocrine hormone influences physiological and circadian processes such as sleep propensity; its synthesis is suppressed by light, particularly in the short-wavelength blue range (Brainard et al. 2001) and transmitted via neural pathways involving the suprachiasmatic nuclei and the autonomic nervous system innervating the pineal gland. The combination of altered environmental light conditions and aberrant melatonin signalling might influence onset and course of BD (Etain et al. 2012). Peaks for both manic and depressive episodes appear to follow a seasonal pattern (Geoffroy et al. 2014) and BD patients’ hypersensitivity of melatonin suppression in response to nocturnal light led to the postulation of supersensitivity to light as a trait marker in BD I patients (Hallam et al. 2009; Lewy et al. 1985; Nurnberger et al. 2000). However, result could not always be replicated and studies on altered melatonin secretion in BD or populations at risk were heterogenous in design and results. Some indicate a phase advance in mania (Nováková et al. 2015), while other studies showed a phase delay in bipolar depression (Robillard et al. 2013; Fang et al. 2021) and in euthymia (Nurnberger et al. 2000; Fang et al. 2021). The dim light melatonin onset (DLMO) is computed after a serial sampling of plasma or saliva melatonin concentration. It is defined as the time the melatonin concentration rises above a certain threshold and usually precedes the accustomed bed time for around 2–3 h and its use as a measurement of circadian phase is recommended by the Chronobiology Task Force of the International Society for Bipolar Disorders (ISBD) (Murray et al. 2020). The phase angle difference (PAD) is a measurement of circadian alignment of different biological rhythms such as melatonin and sleep (Lewy 2009). The circadian rhythm is also governed by cortisol, which is produced in the suprarenal cortex and regulated by the hypothalamic–pituitary–adrenal axis (Dickmeis 2009). BD patients had higher cortisol levels in the dexamethasone suppression test compared to healthy controls, cortisol level correlated positively with the number of previous episodes (Fries et al. 2014) and BD patients with more affective episodes showed reduced cortisol reactivity to stress compared to a subset of patients with less episodes (Havermans et al. 2011). Thus, sleep should be considered as a treatment target throughout all stages of the bipolar illness. The altered internal timing has been targeted by medications used to treat BD (antidepressants, mood stabilizers) and the starting point of the developing of chronobiological therapies (Bellivier et al. 2015; Martynhak et al. 2015). Recently, the six most commonly used adjunctive chronotherapeutic interventions in the treatment of BD (bright light therapy, dark therapy, sleep deprivation, melatonergic agonists, cognitive behavioural therapy for insomnia, and interpersonal social rhythm therapy) have been systematically reviewed. Interventions have been shown to be mostly useful in acute mania or bipolar depression, and light therapy had the strongest evidence base in the acute treatment of bipolar depression (Gottlieb et al. 2019).

Evidence of changes in sleep and circadian alignment during aging such as a shortened TST, a prolonged WASO, worse SE, earlier sleep onset (Yoon et al. 2003; Ohayon et al. 2004) and an absolute and relative advance of DLMO and therefore a smaller PAD (Yoon et al. 2003; Duffy et al. 2002) have been present for some time (Li et al. 2018).

The present cross-sectional study investigates parameters of sleep in a sample of euthymic patients with a diagnosis of bipolar disorder in comparison to healthy control subjects. Serial salivary melatonin sampling (to compute the DLMO), ratings measuring subjective sleep quality and actigraphy were employed as proposed by the ISBD Chronobiology Task Force (Murray et al. 2020) and correlations with clinical and sociodemographic features were assessed.

## Methods

### Participants

52 individuals were recruited, 26 of whom had a diagnosis of bipolar disorder. Subsequently, seven participants had to be excluded because of missing actigraphy data. 45 participants remained (20 males, 36.24 years old), 21 of them suffering from BD. All patients were in clinical remission and underwent out-patient treatment in the LWL-University Hospital Bochum. The control subjects were recruited among students and employees of the Ruhr-University. Structured Clinical Interviews for DSM-V (American Psychiatric Association 2013) confirmed the clinical diagnoses of BD in the patient group and the absence of bipolar symptoms in the control group. The current absence of major manic or depressive symptoms was confirmed by the Hamilton Depression Scale with a score of 17 or higher indicating depression (Hamilton 1960) and the Young Mania Rating Scale with a score of 12 or higher indicating (hypo-) mania (Young et al. 1978) Exclusion criteria comprised acute suicidality, severe (comorbid) psychiatric disorders, incapacity to complete the self-administered questionnaires, and retracted or denied informed consent. Written informed consent was obtained from all participants and the study was approved by the Ethical Committee of the Department of Medicine at the Ruhr- University Bochum (No. 17–6083).

### Instruments

#### Actigraphy

Actigraphy is a valid type of TST, WASO and SE measurement, particularly in outpatients (Martin and Hakim 2011) and has shown comparable results to polysomnography and sleep diaries in BD (Kaplan et al. 2012). The ISBD Chronobiology Task Force recommends the use of Actigraphy as a measurement sleep in BD (Murray et al. 2020). Participants wore a triaxial accelerometer (GENEactiv, Activinsights Ltd., Kimbolton UK) on their non-dominant wrist for seven days. Devices were set to a recording frequency of at least 60 Hz. Raw data were extracted with GENEactiv PC software, processed and analyzed with the GGIR version 1.10-7 for R version 3.6.1 (Hees et al. 2015; Migueles et al. 2019). Raw accelerometric data were analyzed by using a heuristic method processing the collected data in bouts of activity and inactivity depending on the level of movement and change of angle of the wrist at which the device is worn. As sleep is characterized generally by the absence of greater body movements, the software counted bouts of inactivity as “sleep” at all times the participant indicated being in bed. The following values were analyzed: sleep onset, sleep offset, TST, time spent in bed (TIB), sleep efficiency (SE: TST/TIB) and WASO.

#### Pittsburgh sleep quality index (PSQI)

The PSQI is a 19-items-scale to assess subjective sleep quality within a 4-week time frame (Buysse et al. 1989). A total PSQI score (range 0–21) as well as seven subscores (range 0–3) were calculated, with a higher score indicating worse subjective sleep quality. There is 93.4% sensitivity and 100% specificity, with a cut-off PSQI score of > 6 distinguishing between good and bad sleepers (Backhaus et al. 2002 Sep). The internal consistency varies between α = 0.83 and 0.85 for the English and German version respectively (Buysse et al. 1989; Backhaus et al. 2002).

#### Regensburg insomnia scale (RIS)

The RIS is a 10-items-scale to assess cognitive and emotional sleep aspects, as well as insomnia symptoms within a 4-week time frame. A total score (range 0–40) ≥ 13 indicates distinct and ≥ 24 striking psychophysiological insomnia symptoms. The internal consistency is α = 0.89 (Crönlein et al. 2013).

#### Sleep diary

This diagnostic sleep diary consists of questions regarding bedtime in the evening, SOL, frequency and duration of waking phases, time of awakening and rising time. Furthermore, patients’ self-rate their relaxation, capacity and exhaustion in the evening, recovery function of sleep, mood in the morning, alcohol consumption, and the frequency and duration of day sleep (Hoffmann et al. 1997).

#### Melatonin and cortisol

Participants were instructed to take five salivary samples on the last night and a sixth sample the subsequent morning. The evening samples were used to calculate the DLMO and had to be taken four hours before the median sleep onset time in dim light (i.e., no bright light, no direct light to the face). The sixth sample was taken 30–60 min after awakening (Cortisol awakening response). Participants stored the samples in their fridge; samples were centrifuged at 1250*g* and refrigerated at − 20 °C immediately after return. Melatonin was measured in the five evening saliva samples using a competitive enzyme-linked immunosorbent assay (ELISA) with a lower detection level of 1 pg/ml. Inter- and intra-assay coefficients of variation were 7.6–13.0% and 6.1–10.8%, respectively. Morning cortisol levels were measured in morning salivary samples using an electrochemiluminescence immunoassay (ECLIA) with a lower detection level of 0.054 µg/dl and inter- and intra-assay coefficients of variation of 2.5–14.8% and 1.7–9.3%, respectively.

To measure the circadian phase of melatonin we used the DLMO, which is defined as the time at which the melatonin level began to rise above a certain threshold while kept under dim light conditions. The melatonin level had to either exceed 3 pg/ml. If this threshold was not reached, we did conduct a visual inspection of the concerning sample curve and did determine a DLMO if a clear rise of concentration was visible. Salivary melatonin sampling and estimation of DLMO have been recognized as a valid measurement of DLMO in BD by the Chronobiology Task Force of ISBD (Murray et al. 2020). The first rise above that threshold was taken as DLMO time. If the melatonin level in the first sample was already above 3 pg/ml, we assumed the time of our first sample to be the DLMO. To assess the circadian alignment of individuals we computed the phase angle difference (PAD), which is the difference between mid-sleep and the DLMO (Lewy 2009). Since their melatonin samples could not be processed (lack of sample material or no detected rise in melatonin levels, see below), n = 3 participants (2 healthy controls, 1 BD patient) were excluded from further analysis of PAD and DLMO, leaving a cohort size of 22 healthy controls vs. 20 BD patients. Three different individuals did not manage to deliver sufficient cortisol samples and therefore those participants (1 healthy control, 2 BD patients) were excluded from any further cortisol analysis.

### Statistical analysis

Statistical analyses were performed with SPSS^®^ 26.0 for Windows software (IBM). To analyse the study cohort, descriptive statistical methods were used. Quantitative data are presented as mean and standard deviation. For comparison of categorical and continuous variables, chi square tests, unpaired t-tests, and Mann–Whitney U test were used where appropriate. A p-value of less than 0.05 was interpreted as significant. Univariate analysis of covariance (ANCOVA) was conducted for age and gender, given the significant group differences to control for the influence of age and gender. For all analyses, *p* < 0.05 was required for significance.

## Results

### Study population

BD (14 males, 45 ± 12.60 years) and control group (6 males, 28.58 ± 9.58 years) differed significantly in age (*p* < 0.001) and gender (*p* = 0.005) (Table 1). Patients with had a significantly higher BMI (*p* = 0.001) and had more close relatives with SD (38.1% vs. 16.7%) compared to healthy controls. All healthy controls, but only 38.1% of the BD group were employed at time of inclusion, none of them working shifts. The mean age of onset of the first mood symptoms was 24.10 ± 9.75 years, but mean age of first diagnosis was 33.66 ± 11.03 years in BD patients. 95.2% of the BD patients had experienced inpatient care before (5.14 ± 3.72 times). Almost all patients (95.2%) received psychotropic pharmacotherapy; every other (53.4%) reported a history of suicidal behaviour; and every third (33.3%) had attempted suicide before. Their average HAMD-17 score was 4.38 and the average YMRS score was 1.9, thus excluding a current major episode.

**Table 1:** Sociodemographic, clinical and psychopathological characteristics of the two groups investigated

	Healthy controls (N = 24)	Bipolar disorder (N = 21)	*p* values
Female gender (n, %)	18 (75)	7 (33.34)	0.005
Age (years; mean ± SD)	28.58 (SD = 9.58)	45 (SD = 12.59)	< 0.001
Height (cm; mean ± SD)	170.83 (SD = 7.01)	170.67 (SD = 10.37)	0.94
Weight (kg; mean ± SD)	68.58 (SD = 14.60)	84.52 (SD = 19.83)	0.003
Occupation	24 employed (0% unemployment)	8 employed, 13 unemployed (61.9% unemployment)	
Pre-existing endocrine disease	8.3% (*n* = 2)	47.6% (*n* = 10)	
	Hypothyroidism (*n* = 2; 8.3%)	Hypothyroidism (*n* = 9; 42.9%)	
		Diabetes mellitus (type 2) (*n* = 3; 14.3%)	
RLS symptoms	8.3% (*n* = 2)	9.5% (*n* = 2)	
Regularly taken medication	54.2% (*n* = 13)	95.2% (*n* = 20)	
	Oral contraceptives (*n* = 11; 45.8%)	Mood stabilizers (*n* = 20; 95.2%)	
	Antihistamines (*n* = 3; 12.5%)	Antidepressants (*n* = 7; 33.3%)	
	l-Thyroxine (*n* = 2; 8.3%)	Sedative drugs (*n* = 7; 33.3%)	
	Isotretinoin (*n* = 1; 4.2%)	l-thyroxine (*n* = 9; 42.3%)	
	Triptan (*n* = 1; 4.2%)	Antihypertensive drugs (*n* = 7; 33.3%)	
	NSAID (*n* = 1; 4.2%)	Proton pump inhibitors (PPI) (*n* = 4; 19%)	
	ß-Blocker (*n* = 1; 4.2%)	Antidiabetics (*n* = 3; 14.3%)	
		Aspirin (*n* = 1; 4.8%)	
HAMD-17 average score	3.00	4.38	0.036
YMRS average score	2.00	1.90	0.847
PSQI average score	3.63	5.52	0.022
RIS average score	5.83	8.90	0.031

NSAID, non-steroidal anti-inflammatory drug; RLS, restless legs syndrome

### Subjective sleep reception (PSQI, RSI and sleep diary)

BD group and healthy controls differed significantly in the PSQI (5.52 ± 3.14 vs. 3.63 ± 2.18; *p* = 0.022) and in the RIS (8.91 ± 5.43 vs. 5.83 ± 3.76; *p* = 0.031). Despite these differences, none of the groups reached a mean score high enough to indicate overall bad sleep. On an individual level, six BD patients and two healthy controls reached the PSQI cut- for bad sleep and five BD patients as well as one healthy control reported distinct psychophysiological symptoms of insomnia according to RIS. According to the sleep diaries, patients had a longer subjective TST (*p* = 0.024), and TIB (*p* = 0.002),

### Actigraphy

As in Table 2 presented, BD group showed in comparison to healthy controls a longer WASO (72.90 ± 35.86 vs 51.56 ± 16.77; *p* = 0.019) had a worse SE (84.45% ± 6.75% vs. 88.01% ± 3.30%; *p* = 0.036), fell asleep earlier (23.37 ± 1.16 vs. 24.35 ± 0.79; *p* = 0.002) and sleep onset time varied less (0.95 ± 0.59 vs. 1.47 ± 0.59; *p* = 0.005), which could indicate a more stable sleep schedule. There were no significant differences for actigraphically validated TST.

**Table 2:** Group differences for the main outcome variables

	Healthy controls	Bipolar disorder	*t*-test
TST (sleep diary)	437.51 (SD = 52.52)	478.28 (SD = 64.64)	*p* = 0.024
TIB (sleep diary)	486.26 (SD = 58.75)	550.89 (SD = 73.83)	*p* = 0.002
TST (actigraphy)	402.63 (SD = 47.37)	428.05 (SD = 71.59)	*p* = 0.176
WASO (actigraphy)	51.56 (SD = 16.77)	72.90 (SD = 35.86)	*p* = 0.019
SE (actigraphy)	88.01% (SD = 3.30%)	84.45% (SD = 6.75%)	*p* = 0.036
Sleep onset (actigraphy)	24.35 (SD = 0.79)	23.37 (SD = 1.16)	*p* = 0.002
Average melatonin	3.35 (SD = 2.23)	4.06 (SD = 2.77)	*p* = 0.352
Cortisol	17.06 (SD = 5.37)	16.97 (SD = 10.22) *n* = 18	*p* = 0.969
DLMO	21.82 (SD = 1.50) n = 22	20.17 (SD = 1.63) *n* = 20	*p* = 0.001
Phase angle difference	6.35 (SD = 1.40) *n* = 22	7.48 (SD = 1.53) *n* = 20	*p* = 0.017

### Melatonin and cortisol

**Fig. 1:**
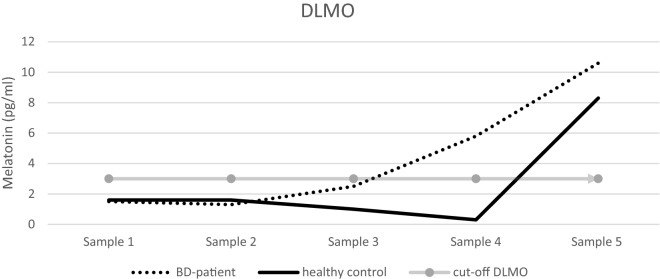
Melatonin curves of bipolar versus healthy subjects. DLMO in BD patients was on average 1.65 h earlier than in healthy controls

There was no significant difference in average melatonin levels between BD patients and healthy controls (4.06 ± 2.77 vs. 3.35 ± 2.23 *p* = 0.352). The DLMO was on average 1.65 h earlier in BD patients in comparison to healthy controls (20.17 ± 1.63 vs. 21.82 ± 1.50; *p* = 0.001). When computing the PAD to quantify possible circadian misalignment, we found a significant phase advance of melatonin in BD patients compared to healthy controls (6.35 ± 1.40 vs. 7.48 ± 1.53; *p* = 0.017). Two examples of melatonin curves are displayed in Fig. 1.

There was no significant difference between the mean cortisol levels 30 min after rising time in both groups. The average sampling time was at 8:08 am (SD = 1:24) for controls and at 8:17 am (SD = 2:05) for BD. Three individuals were excluded from analysis: two had strikingly low cortisol levels that was attributed to sampling error and one failed to deliver enough saliva. All of them were part of the BD-group.

### Correlation of main outcome variables

A larger PAD was highly correlated with longer WASO, a worse SE and a longer duration of illness (WASO: r = 0.676, p = 0.001; SE: r = − 0.554, p = 0.011; duration of illness: r = 0.458, p = 0.042). Concurrently we found a correlation between an earlier DLMO and a higher WASO as well as a longer duration of illness (WASO: r = − 0.467, p = 0.038; duration of illness: r = − 0.583, p = 0.007). Longer time passed since the last episode experienced was highly correlated to higher cortisol levels (r = 0.796; p = 0.000077) and lower TST (r = − 0.438; p = 0.047). Longer time since the last manic episode was associated with a worse SE and a lower TST both in actigraphy and sleep diary (SE: r = − 0.516, p = 0.02; TST (actigraphy): r = − 0.559, p = 0.01; TST (sleep diary): r = − 0.486, p = 0.03). We found age at first diagnosis to be negatively correlated with SE (r = − 0.453; p = 0.039). We also found a correlation of between a higher score in HAMD-17 and a higher score in PSQI as well as in RIS (PSQI: r = 0.514, p = 0.017; RIS: r = 0.615, p = 0.003). There were no correlations between any sleep outcome variable and YMRS scores, age at first phase and number of depressive episodes.

### Influence of age and gender

Group differences in PSQI (F = 4.85; p = 0.03), subjective TST (F = 4.36, p = 0.043) and subjective TIB (F = 6.12, p = 0.018) remained significant after controlling for age and gender, while group differences in RIS (F = 2.83; p = 0.1), actigraphically measured sleep onset (F: 3.971; p = 0.053), SD of said sleep onset (F: 1.218, p = 0.276), WASO and SE (WASO: F = 1.252, p = 0.27; SE: F = 0.529, p = 0.471), DLMO (F = 2.11; p = 0.155) and PAD (F = 1.460; p = 0.234) did not. DLMO, PAD and actigraphically measured WASO and SE remained significant, when we only controlled for the covariate gender, while there developed a trend towards a longer TST in our BD group when we controlled for the covariate age (F: 3.261; p = 0.072). This trend was further strengthened by also controlling for gender (F: 3.999; *p* = 0.052).

## Discussion

In comparison to healthy controls, BD patients had: (1) higher PSQI (significant after controlling for age and gender), and higher RIS scores; (2) subjective a longer mean TST and TIB (both significant after controlling for age and gender), longer WASO, and worse SE (significant after controlling for gender); (3) actigraphically validated earlier sleep onset, less variation in sleep onset time, and no longer TST; (4) no differing melatonin levels, an 1.65 h earlier DLMO, and a phase advance of melatonin (both significant after controlling for gender); and (5) no differing cortisol awakening response.

Individuals suffering from BD had significantly higher PSQI and RIS score. Those findings of subjectively worse sleep are also a common feature of interepisode BD (Ng et al. 2015). There were more patients than healthy controls, who had a PSQI- or RIS-score, which was high enough to indicate symptoms of low sleep quality (PSQI) or insomnia (RIS). Although this difference remained a statistical trend, it further strengthens our findings of worse subjective sleep quality in BD, which are in harmony with several previous reports worse subjective sleep quality as a feature of interepisode BD (Cour Karottki et al. 2020).

This study confirms that euthymic BD patients have a prolonged total sleep time—at least in their self-rating. Actigraphy did not confirm those impressions. Although Ng et al. reported different results in their meta-analysis there are several meta-analysis which report longer TST in actigraphy studies (Tazawa et al. 2019; Ng et al. 2015; Meyer et al. 2020). There is some evidence for a disturbed perception of sleep and therefore an overestimation of TST in BD, which our results would confirm as well (Krishnamurthy et al. 2018). Nonetheless this study was conducted in symptomatic BD patients and several studies reported either no differences in subjective and objective TST estimation (Fujita et al. 2021), an overestimation of subjective TST compared to objectively measured TST, which did not differ between BD and healthy participants (Ihler et al. 2020) or an underestimation of TST in BD compared to healthy controls (Ritter et al. 2016).

However our results of a longer subjective TIB are consistant with a meta-analysis of actigraphic studies (Meyer et al. 2020).

This study confirms that euthymic BD patients have more WASO and a worse objective sleep quality measured in SE. These results match previous actigraphic findings of longer WASO and a subsequently worse SE in euthymic BD (Meyer et al. 2020; Tazawa et al. 2019). However, when analysing our actigraphic data we did not find any significant difference in TST between both groups, which stands in contrast to several meta-analysis of actigraphically derived data in BD (Tazawa et al. 2019; Geoffroy et al. 2015; Meyer et al. 2020; Crescenzo et al. 2017). Our findings of an earlier sleep onset and a smaller SD in sleep onset in our BD group contradict previous studies of chronotype and circadian rhythm in BD, which showed a later chronotype to be associated with BD diagnosis (Ahn et al. 2008; Wood et al. 2009) Although those changes might be associated with more mood symptoms and therefore a potential worse clinical outcome (Krane-Gartiser et al. 2016; Vidafar et al. 2021), they were not as consistently shown in inter-episode states of BD, since Krane-Gartiser et al. (2016) found delayed sleep phase syndrome in only about 25% of all patients and Vidafar in not more than 50% with the remaining patients having either a normal or early chronotype. Also there are methodological differences between those studies and our study in assessment of chronotype.

In our study, no significant difference in average melatonin concentration was found, which contradicts the findings of a previous study that found lower melatonin secretion to be a possible trait marker for BD (Kennedy et al. 1996). This contradiction might be explained through our shorter sampling window, which did not include any samples after each individual’s median bedtime.

The detection of an earlier DLMO and a greater phase angel difference is new and somewhat contrary to previous findings. Early approaches from Kripke et al. (1978) to study circadian rhythms in BD also found phase advances in several circadian rhythms. Overall, recent results in this field of study are mixed due to different study designs and focus; our study might be the first to focus on euthymic BD patients and to use DLMO and PAD to determine circadian alignment. Other studies using some kind of melatonin measurement found: (a) a phase delay in patients with bipolar depression (Robillard et al. 2013; Fang et al. 2021); (b) a significantly later peak in melatonin concentration in euthymic patients suffering from BD subtype I (Nurnberger et al. 2000); (c) a possible phase advance in manic individuals with BD, with significantly higher afternoon levels compared to individuals with bipolar depression or healthy controls (Nováková et al. 2015); and (d) no difference in phase between euthymic bipolar patients and healthy controls (Fang et al. 2021). Contradictions of our results to those of Nováková et al. (2015), Fang et al. (2021) and Robillard et al. (2013) might be explained by mood state, since we only included euthymic BD patients and—in the case of Fang et al. (2021)—recruited a larger euthymic BD group. With regard to Nurnberger et al. (2000), we did not differentiate between subtypes of BD because of our small sample size, and our sample of BD patients only included one individual who did not receive any psychotropic medication. Furthermore, we examined PAD and DLMO instead of peak time of melatonin. Hoyos et al. (2020) found similar alterations of DLMO and PAD in a subset of elderly patients with major depressive disorder (MDD). These similarities are surprising, thus melatonin secretion and its features, such as hypersensibility to suppression by light, have been discussed to be a trait marker of BD or for distinguishing BD from MDD (Lewy et al. 1985; Nurnberger et al. 2000; Lewy et al. 1981; Nathan et al. 1999), although several studies showed that melatonin secretion in BD and MDD could not be differentiated by their reaction to light (Lam et al. 1990; Whalley et al. 1991). An earlier DLMO and a subsequently larger PAD as a feature of BD could help to further understand the therapeutic effects of mood stabilizers, as lithium has been shown to delay several circadian rhythms, such as REM sleep and the acrophase of core body temperature (Campbell et al. 1989). Although there are studies suggesting that neither lithium nor sodium valproate (common mood stabilizers in the treatment of BD) directly delayed DLMO, both did show a significant reduction in the hypersensitivity of melatonin secretion to light (Hallam et al. 2005a, b).

We found no significant differences between awakening cortisol levels of BD patients and healthy controls. This result conflicts with findings of prior research in this field, which found higher levels of morning cortisol in BD patients (Girshkin et al. 2014; Belvederi Murri et al. 2016). Explanations for differences between our results and those of prior research might include a smaller sample size and the inclusion of only euthymic bipolar patients, as well as the fact that we did not specify a certain sampling time, allowing for a greater variance in the sampling times.

When looking only at our BD sample we found that age at first diagnosis was negatively correlated with SE, which could indicate an earlier diagnosis and thus treatment of BD would help to improve the future sleep functioning of patients. This relationship between early diagnosis and a better sleep outcome further underlines the importance of early recognition of symptoms and treatment of BD. We found significant correlations between a larger PAD and a worse SE, longer WASO and longer duration of illness. These results do indicate a link between a worse sleep outcome in BD and a circadian misalignment of melatonin and sleep–wake rhythm. The association between a larger PAD and a longer duration of illness might indicate that the misalignment of circadian rhythms progresses throughout the course of BD. The simultaneously found correlations between an earlier DLMO and a longer WASO and longer duration of illness are on the one hand logical consequence of our methods since we used DLMO to calculate PAD but on the other hand strengthen the implications of our results.

Longer time since the last episode was highly correlated to a higher rising cortisol level and also correlated to a shorter TST. These results as well as the correlation between longer time since the last manic episode and shorter TST and worse SE might indicate that time since the last (manic) episode might not be a measurement to test for disease stability and could even be associated with a higher risk of relapse, thus higher cortisol levels and shorter TST are both features of mania in BD (Kaplan et al. 2012; Cervantes et al. 2001). Additionally, it was shown that sleep disturbance precedes the onset of mood episodes for other affective disorders, such as unipolar depression, and shorter sleep time predicts worsening of depressive symptoms over a six-month period (Perlis et al. 1997; Perlman et al. 2006). However, we found no correlations between TST, SE or rising cortisol levels and HAMD-17 or YMRS scores, indicating a prodromal status for either polarity.

Our findings of correlation between higher HAMD-17 and worse sleep quality or more insomnia symptoms measured by PSQI and RIS, correspond to findings of associations between PSQI and depressive symptoms in larger samples (Huang and Zhu 2020). Although none such association has been established for RIS, we assume that a similar relationship might also explain this correlation of RIS-score and HAMD-score.

Due to the naturalistic setting of this study and several drop outs, because of unretrievable or insufficient actigraphy data our BD and control group differed in age and gender, giving us the opportunity and obligation to control and further elaborate the influences of those covariates. While almost all differences in subjective sleep variables except for RIS scores were unaffected by influences of age and gender, the group differences in our other variables might have been influenced by those covariates. Especially age seems to have had an impact on objective sleep variables (WASO, SE, actigraphic sleep onset and SD of sleep onset) and circadian variables (PAD, DLMO), since group differences in these variables remained significant when only controlling for gender. The finding of a worse sleep quality with more WASO and a subsequently worse SE in aging populations is consistent with previous meta-analysis (Ohayon et al. 2004; Jonasdottir et al. 2021). The influence of age on the group differences in actigraphically measured sleep onset and SD in said sleep onset helps to encorporate our results in previous findings (Ahn et al. 2008; Wood et al. 2009) as contradictions are surpsining and there is evidence of our chronotype and sleep onset changing and becoming earlier over the course of adolescence and adulthood (Yoon et al. 2003; Logan and McClung 2019). In regards to PAD and DLMO there are previous results of an earlier DLMO during aging (Yoon et al. 2003; Duffy et al. 2002), which might help to understand the advance of DLMO in our cohort also as a result of age and not only BD. Against this background the loss of significance of the PAD group difference in ANCOVA for age is somewhat unexpected, as a longer PAD was found (BD group) and aging in previous studies has had a shortening effect on PAD (Yoon et al. 2003; Duffy et al. 2002). This might indicate an inverse effect of age on PAD in BD.

Interestingly TST also shortens during aging (Ohayon et al. 2004; Jonasdottir et al. 2021), which might explain the absence of a longer actigraphically validated TST in our BD group in spite of large evidence for this sleep alteration in interepisode BD (Meyer et al. 2020). While controlling for age as covariate did not lead to a significant group difference in TST, it revealed a trend, which might help explain our conflicting results concerning a usually common finding of longer TST in interepisode BD.

Although there is evidence for an more WASO (Ohayon et al. 2004; Jonasdottir et al. 2021), an earlier sleep onset and a lower variability of sleep onset (Jonasdottir et al. 2021) in women, our group differences in those variables did not change when controlling for gender. Since results concerning the influence of gender on TST are conflicting with some reporting a longer TST in women (Jonasdottir et al. 2021), while others saw longer TST in men (Ohayon et al. 2004) it might be of interest to note, that the level of significance for our group difference in TST was raised when controlling not only for age but also for gender. In spite of several studies reporting an earlier DLMO (Titone et al. 2020; Cain et al. 2010; Burgess and Eastman 2005; Reen et al. 2013) and a larger PAD (Cain et al. 2010; Burgess and Eastman 2005; Reen et al. 2013) in women our results of DLMO and PAD remained the same after controlling for gender.

There are several limitations to our study. Firstly, our study had a naturalistic design and several drop outs due to unsufiicient actigraphic data. In consequence there were group differences in age and gender and a smaller then expected group size, which might have influenced our results. Secondly, it is important to recognize that our sampling window for DLMO was rather short due to the high cost of melatonin analysis. Although we could identify most individuals’ DLMO in our sampling window, there were some cases where an individual’s first sample was already above our DLMO threshold, which forced us to assume this person’s DLMO. A higher sampling frequency and a larger sampling window would be worthwhile. Thirdly, although subjects received detailed, easy-to-understand information, they had to retrieve saliva samples on their own at home, which might have hampered the results due to non-compliance or misunderstanding. Lastly, our sample size was rather small, so our findings would need to be confirmed in a larger setting and—until then—must be interpreted with great caution.

## Conclusions

We found that sleep in interepisode BD is perceived as significantly worse. Although objective sleep variables did not remain significant throughout ANCOVA, there was a trend towards a significantly longer TST and patients showed a lower SE and higher WASO. Our results hint at a circadian misalignment with an earlier DLMO and a larger PAD in interepisode BD, which might further support Kripke et al. (1978) ’s theories of phase advance in BD. A worse sleep outcome and longer duration of illness were associated with circadian misalignment in our BD sample.

## Data Availability

Data availability on request.

## Abbreviations

ANCOVA: Analysis of covariance
BD: Bipolar disorder
DGSM: Deutsche Gesellschaft für Schlafforschung und Schlafmedizin (German Sleep Society)
DLMO: Dim light melatonin onset
HAMD: Hamilton depression scale
ICD: International Statistical Classification of Diseases and Related Health Problems
ISBD: International Society of Bipolar Disorders
NSAID: Non-steroidal anti-inflammatory drugs
PAD: Phase angle difference
PSQI: Pittsburgh sleep quality index
RIS: Regensburg insomnia scale
RLS: Restless legs syndrome
SD: Sleep disturbances
SE: Sleep efficiency
SOL: Sleep onset latency
TIB: Time in bed
TST: Total sleep time
WASO: Wake after sleep onset
WHO: World Health Organisation
YMRS: Young mania rating scale
